# Do flowers removed of either nectar or pollen attract fewer bumblebee pollinators? An experimental test in *Impatiens oxyanthera*

**DOI:** 10.1093/aobpla/plab029

**Published:** 2021-05-28

**Authors:** Deng-fei Li, Xian-chun Yan, Yi Lin, Li Wang, Qiong Wang

**Affiliations:** 1 Key Laboratory of Southwest China Wildlife Resources Conservation (Ministry of Education), China West Normal University, Nanchong 637002, China; 2 School of Life Sciences, Central China Normal University, Wuhan 430079, China

**Keywords:** Butterfly, dichogamy, honey bee, nectar robbing, pollen thieves, reward, visit duration, visitation rates

## Abstract

Pollen and nectar are the primary rewards offered by flowers to pollinators. In floral visitors of some plant species, pollen thieves and nectar robbers cause the reduction in pollen grain number and nectar volume, respectively. However, it remains unclear whether the absence of either of the two rewards in a given flower reduces its attraction to nectar- and pollen-collecting pollinators. We hypothesized that flowers removed of either nectar or pollen would attract fewer pollinators. We studied protandrous *Impatiens oxyanthera*, whose flowers provide bumblebee pollinators with both nectar and pollen in the male phase. We conducted floral reward manipulation experiments to explore how the removal of either nectar or pollen from flowers influences pollinator behaviour by comparing their visitation rates and visit duration. Compared with the control flowers, the flowers removed of pollen attracted significantly more bumblebee pollinators per 30 min, but the flowers removed of nectar or those removed of both pollen and nectar attracted significantly fewer bumblebee pollinators per 30 min. Moreover, the visit duration of bumblebee pollinators to control flowers or flowers removed of pollen was longer than that to flowers removed of nectar or those removed of both pollen and nectar. Our investigations indicated that compared with control flowers, the flowers removed of nectar attracted fewer bumblebee pollinators, supporting our hypothesis. However, our other hypothesis that pollen removal would reduce pollinator visits was not supported by our results. Instead, compared with control flowers, the flowers that contained only nectar attracted more bumblebee pollinators. Nectar seems to be the main reward, and bumblebee pollinators mainly used the absence of pollen as a visual signal to locate *I. oxyanthera* flowers with a potentially higher amount of nectar.

## Teaser

Pollen and nectar are the primary rewards offered by flowers to bees. Some species’ flowers are protandrous and hide the nectar in a spur and expose the pollen outside in the male phase. And the flowers without anthers (i.e. without pollen) in the female phase have a greater nectar volume. Some bees that mainly forage nectar may use the absence of pollen as a visual signal to locate flowers with a higher amount of nectar.

## Introduction

In many species of flowering plants, sexual reproduction depends on animals for pollination because they cannot produce offspring by spontaneous autogamy or apomixis (Goulson 1999; Vogler and Kalisz 2001; Ratnayake *et al.* 2007; Ollerton *et al.* 2011). Pollen and nectar are the primary rewards offered by flowers to the visiting animals in order to ‘buy’ their services as pollinating agents (Neff 1981; Cook *et al.* 2010; Somme *et al.* 2014; Pyke 2016; Ruedenauer *et al.* 2019). The role of pollen and nectar in attracting potential pollinators has been acknowledged for hundreds of years (Neff 1981). Bees are particularly special flower visitors because, almost uniquely, they use both nectar and pollen as food and completely rely on them for both adult and larval nutrition (Roulston and Cane 2000; Cane *et al.* 2011; Nicolson 2011; Willmer 2011; Somme *et al.* 2014). Adult bees consume nectar and usually some pollen as well, whereas larvae consume large quantities of both pollen and nectar (Andreas 2006; Nicolson 2011; Willmer 2011; Konzmann *et al.* 2019). Thus, bees are collecting these food sources not just for their own needs, but for their offspring as well (Thorp 2000; Kitaoka and Nieh 2009; Somme *et al.* 2014).

In some plant species, flowers offering both of these rewards may attract not only pollinators, but other types of visitors as well, such as pollen thieves (Sébastien and Forrest 2019) or nectar robbers (Ethan and Irvin 2002). While pollinators forage nectar and/or pollen and provide a pollination service, pollen thieves or nectar robbers, due to a morphological mismatch between their bodies and flowers, only acquire pollen or nectar reward without pollinating the plants they visit (Hargreaves *et al.* 2010; Irwin *et al.* 2010).

Previous studies have indicated that pollen theft or nectar robbing may have negative effects on plant fecundity through reducing pollinator attraction (Hargreaves *et al.* 2010; Irwin *et al.* 2010). Firstly, pollen has a direct function in plant mating as the carrier of male gametes, and thus, its removal by pollen thieves represents consumptive emasculation that can directly reduce siring opportunities (do Carmo *et al.* 2004). Pollen can also act as a visual or olfactory pollinator attractant (Dobson and Bergstrom 2000; Lunau 2000; Pernal and Currie 2002), and both nectar- and pollen-collecting pollinators use its absence to identify and avoid flowers that have been visited recently (Dobson and Bergstrom 2000; Lunau 2000). Duffy and Johnson (2011) showed that pollen production could positively affect the fecundity of *Aloe maculata* through increasing pollinator attraction when it was used as a reward for pollinators. Bees were observed to visit emasculated plants less frequently than non-emasculated controls (Duffy and Johnson 2011; Duffy *et al.* 2014). Therefore, flower emasculation may result in the absence of pollination. Secondly, nectar robbers feed on nectar by biting holes in flowers without contact with the anthers and/or stigma (Inouye 1983; Maloof 2000). The reduced nectar volume in the robbed flowers may result in decreased visitation from pollinators (Irwin *et al.* 2010; Nakamura and Kudo 2016; Rojas-Nossa *et al.* 2021). Ethan and Irvin (2002) showed that naturally-robbed male-phase flowers contained one-fifth the amount of nectar found in non-robbed male-phase flowers of *Impatiens capensis*. Dreisig (2012) found that a bee visited more *Anchusa officinalis* flowers per plant when the plant had a certain amount of nectar, whereas it left the unrewarding plant as soon as possible. For self-compatible plants that cannot autogamously self-pollinate as well as for self-incompatible species, reliance on pollinators has a significant effect on the degree to which nectar robbing affects female plant reproduction (Burkle *et al.* 2007, Zhang *et al.* 2009). However, experimental evidence for the effects of absence of either pollen or nectar in a given flower on pollinator attraction remains scarce.

In terms of the two rewards for pollinators (pollen and nectar), visits by pollen thieves can result in a reduction in pollen grains, whereas, nectar robbers cause nectar scarcity. The absence of either of the two rewards in a given flower may reduce flower attractiveness for nectar- and pollen-collecting pollinators. Therefore, we hypothesized that flowers removed of either pollen or nectar would attract fewer pollinators.

To test this hypothesis, we studied *Impatiens oxyanthera*, a plant with flowers characterized by a long and curved spur, on Mt. E’mei located southeast of the Sichuan Basin, China. The studied species provides bumblebee pollinators with both nectar and pollen. We conducted floral reward manipulation experiments to explore how flowers removed of either nectar or pollen influence the behaviour of pollinators by comparing their visitation rates and visit duration. Based on field investigations, we aimed to address the following questions: (i) does the sexual reproduction of *I. oxyanthera* depend on pollinators? (ii) are floral rewards consumed by other types of visitors except for pollinators? and (iii) do flowers removed of either pollen or nectar attract fewer pollinators? This study will be helpful for understanding the adaptations of floral rewards and the role of floral rewards in maintaining relationships between plants and pollinators.

## Materials and Methods

### Study species and site

*Impatiens oxyanthera* (Balsaminaceae) is a hermaphroditic annual herb endemic to south-western China (Chen 2001). It grows in shaded and moist habitats. Its flowers are protandrous and zygomorphic, with three petals and three sepals. Two of the sepals are small and red, whereas the third is enlarged and saccate, and terminates in a nectar-containing spur. A nectary is located at the tip of the nectar spur. The flower has one upper and two lateral, lobed petals. Flowers open in the male phase and contain partially fused stamens (= androecium) covering the stigma. As the anthers dry, the androecium falls off and exposes the stigma, and the flower enters the female phase. The flowers are thus developmentally hermaphroditic, but function with distinct and non-overlapping male and female phases. Individual flowers remain open for 5–7 days. Because of protandry, flowers are in the male phase for 3–4 days and in the female phase for 2–3 days (Wang *et al.* 2013).

We observed the flowers of *I. oxyanthera* in August 2020 on Mt. E’mei, southwest of the Sichuan Basin, China. The observed plants grew on a small sloping meadow located halfway up the mountain (29°34’39”N, 103°16′59″E, and 1241 m above sea level). Bamboo forests and tea trees grew in the studied area.

### Floral reward production and pollination treatments

To evaluate the production of floral rewards (i.e. pollen and nectar) in *I. oxyanthera*, 60 bagged flowers (10 for pollen and 50 for nectar) from different plants were examined on sunny and warm days. Firstly, the anthers of each flower were dissected and washed in 10 mL of distilled water to dislodge the pollen grains from the anthers. The suspensions were stirred in a vortex mixer for 2 min, and five 1-μL samples of suspension were drawn, after which the number of pollen grains in the samples was counted under a light microscope at ×40 magnification. Pollen count of the five sub-samples (1 μL each) was averaged and multiplied by the dilution factor (10 000) to obtain the total number of pollen grains per flower. Secondly, nectar volume was measured using a 10 µL microlitre syringe (Agilent Technologies Inc., USA) in the period of 10:00–11:00 am for male-phase flowers (open 1–3 days, 10 flowers measured each day) and female-phase flowers (open 4–5 days, 10 flowers measured each day) to determine daily variation in nectar volume of *I. oxyanthera*.

To determine the necessity of pollination for sexual reproduction of *I. oxyanthera*, we conducted field experiments on 80 flowers using four pollination treatments. The flowers were randomly selected from different plants to reduce the possible effect of resource reallocation on the fruit set, and they were enclosed in fine-mesh polyester bags to exclude any visitors before the start of the treatments. To test for potential autogamy, 20 flowers were bagged to exclude any insects. In addition, 20 flowers were hand-pollinated with self-pollen grains from flowers of the same individual, and another 20 flowers were hand-pollinated with outcross pollen grains from multiple flowers of other individuals to test for any differences in seed production between selfing and outcrossing. These flowers were bagged again after hand pollination. The remaining 20 flowers without any treatment were exposed to open-pollination as a natural control. Two weeks later, the fruits produced by these flowers were harvested, and the seeds and undeveloped ovules in each fruit were counted.

### Floral reward consumed by various visitors

To estimate the types of floral visitors and their foraging behaviours (for pollen or/and nectar) in *I. oxyanthera*, we observed the foraging behaviours of various visitors in the periods of 08:30–11:30 am and 12:00–17:00 pm on sunny days in August 2020. Two plots (1 × 1 m) were randomly established, each including 20 flowers. These plots were observed daily for a period of 30 min. A total of 66 observation sessions were conducted. Using a camera (Nikon D5300), we recorded the foraging behaviours of various visitors. In addition, we recorded the number of visits and the amount of flowers visited by various visitors per 30 min, after which we calculated the visitation rates of each visitor (visits per flower per 30 min) by dividing the total number of observed flowers by the number of flowers visited per 30 min. The main pollinators were determined by the number of visits and the foraging behaviours. The bees with corbiculae were captured, and the pollen grains in the corbiculae were scratched using a forceps on a microscope slide to determine whether the pollen grains of *I. oxyanthera* were found under the light microscope.

### Floral reward manipulation experiments

To compare the visitation rates and visit duration of pollinators to flowers removed of either pollen or nectar, more than 100 floral buds were selected randomly from different individuals, and bagged with fine-mesh polyester bags before blooming in August 2020. When the flowers were in bloom, we conducted field experiments on 80 male-phase flowers by setting four floral reward treatments before 8:00 am each day. The experiments were as follows: (i) control: 20 flowers without any treatment as a natural control; (ii) pollen removed: 20 flowers were removed of anthers using a forceps. For this treatment, we used male-phase flowers instead of female-phase flowers to make sure that the floral reward (nectar) is at the same level as that in the control treatment, even though female-phase flowers did not have anthers; (iii) nectar removed: 20 flowers were removed of nectar by inserting a 10-µL syringe into each flower at the junction between the nectar-containing spur and saccate sepal to remove all nectar following the methods described in Ethan and Irvin (2002). For this treatment, we removed all nectar to mimic nectar-robbed flowers. All nectar was removed because the remaining amount of nectar in a flower after it is robbed in nature is often too low to even measure it; and (iv) pollen and nectar removed: 20 flowers were removed of both pollen and nectar using procedures as in treatments 2 and 3. The last treatment was for reference. All flowers were exposed to pollinator visits after the treatments. Subsequently, the visitation rates of pollinators (visits per flower per 30 min) were recorded in the periods of 08:30–11:30 am and 12:00–17:00 pm, and their visit duration was recorded randomly. A total of 380 flowers were used for the four floral reward treatments, and a total of 130 h of observations were conducted.

### Statistical analyses

For the pollination treatments, a generalized linear model (GLM) with binomial distribution and logistic-link function was used to detect the effects of the treatments on seed set (with seed number as event variable, total ovule number as trial variable and different treatments as factors) and fruit set (with fruit number as event variable, total treated flower number as trial variable and different treatments as factors). We also performed a GLM with normal distribution and identity-link function to test for differences in the visitation rates of various visitors (with visitation rate as dependent variable, and visitor types as factors). In the floral reward manipulation experiments, a GLM with normal distribution and identity-link function was conducted to identify differences in the visitation rates (with visitation rate as dependent variable, and different treatments as factors) and visit duration (with visit duration as dependent variable, and different treatments as factors) of pollinators to flowers among four treatments. All statistical analyses were performed in SPSS V. 19.0 (SPSS Inc., USA).

## Results

### Floral reward production and pollination treatments

**Figure 1.**
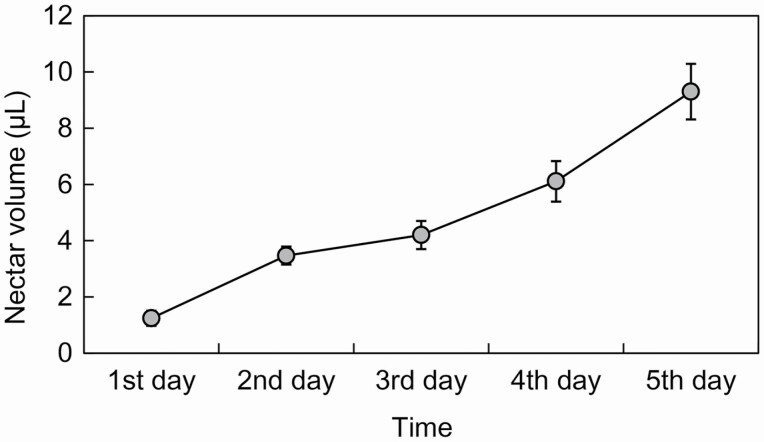
Daily variation in nectar volume of *Impatiens oxyanthera*.

On average, each *I. oxyanthera* flower produced 246 800 ± 11 140 (mean ± SE, *N* = 10) pollen grains. The nectar volume was increasingly secreted with the progress of time during the flowering phase (Fig. 1). The nectar volume of flowers increased from 1.24 ± 0.27 μL (mean ± SE, *N* = 10, Fig. 1) on the first day to 4.2 ± 0.5 μL (mean ± SE, *N* = 10, Fig. 1) on the third day in the male phase and from 6.11 ± 0.72 μL (mean ± SE, *N* = 10, Fig. 1) on the fourth day to 9.3 ± 0.99 μL (mean ± SE, *N* = 10, Fig. 1) on the fifth day in the female phase.

**Figure 2.**
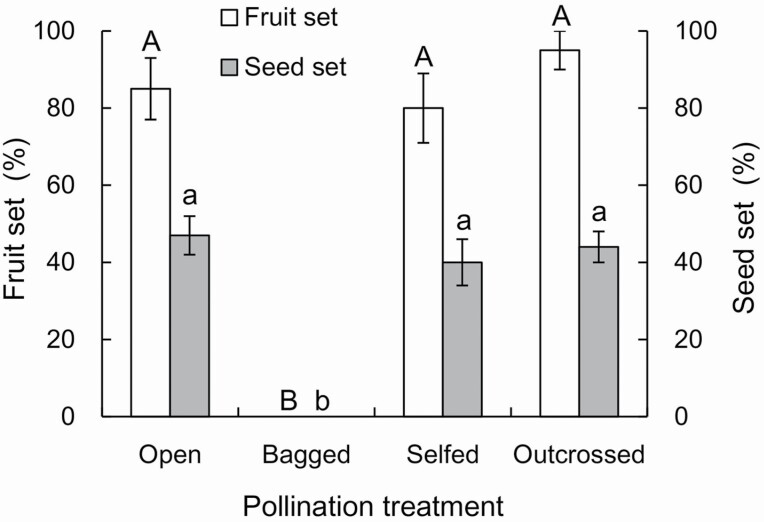
Pollination treatments of *Impatiens oxyanthera*. Different letters above the bars show significant differences at *P* < 0.001 (GLMs). Open, open-pollination; Bagged, bagged without any treatment; Selfed, self-pollination; Outcrossed, cross-pollination.

The effects of pollination treatments on the sexual reproduction *I. oxyanthera* are shown in Fig. 2. Under natural conditions, the fruit set was 85 ± 8.2 % (*N* = 20). There was no significant difference in the fruit set between hand self-pollination (80 ± 9.2 %, *N* = 20) and hand cross-pollination (95 ± 5 %, *N* = 20), indicating that *I. oxyanthera* is highly self-compatible (Fig. 2). There was no significant difference in the seed set between open-pollination (47 ± 5.13 %, *N* = 17) and cross-pollination (44 ± 3.63 %, *N* = 19), showing that there is no pollen limitation under natural conditions (Fig. 2). The fruit set rate in the bagged flowers without any treatment was zero (*N* = 20), which indicated that neither spontaneous autogamy nor apomixis occurs in this species.

### Floral reward consumed by various visitors

**Figure 3.**
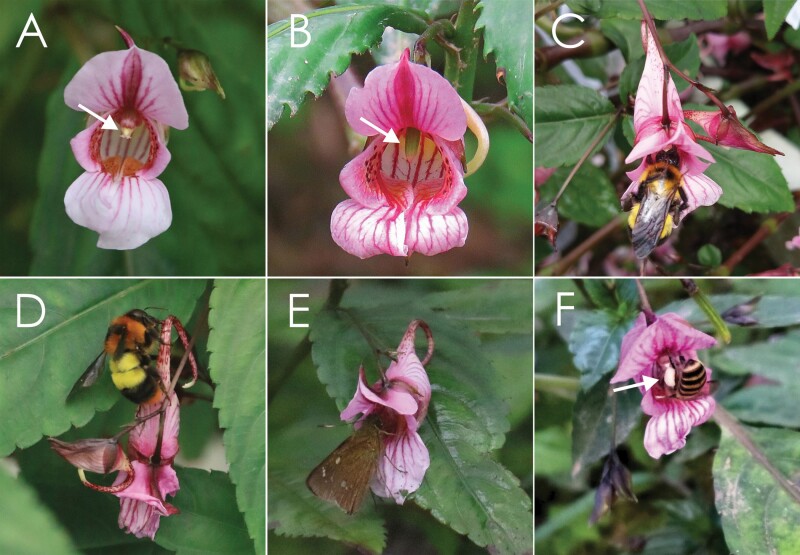
Diverse insects visiting flowers of *Impatiens oxyanthera*. (A) A male-phase flower, the stamens marked with a white arrow. (B) A female-phase flower, the style marked with a white arrow. (C) A pollinator *Bombus trifasciatus* (Apidae) entering corolla and foraging nectar, the thoracic dorsum and wings deposited with a lot of white pollen. (D) A nectar robber *Bombus breviceps* (Apidae) obtaining nectar via a hole bitten on the nectar spur. (E) A butterfly entering corolla and sucking nectar. (F) A honey bee holding stamens and collecting pollen, a lot of white pollen in its corbiculae marked with a white arrow.

Four visitor species were observed visiting the flowers of *I. oxyanthera* to forage for floral rewards during our observations. The GLM analysis showed that there were significant differences in the visitation rates among the four visitor species (Wald χ^2^ = 109.443, df = 3, *P* < 0.001, Table 1). The two most common visitors were *Bombus trifasciatus* (54.301 %) and *Bombus breviceps* (32.796 %), with the mean visitation rates of 0.401 ± 0.042 and 0.212 ± 0.039 visits per flower per 30 min, respectively (mean ± SE, *N* = 66, Table 1). The remaining 12.903 % of the total visits were made by butterflies (0.044 ± 0.014 visits per flower per 30 min, *N =* 66, Table 1) and honeybees (0.008 ± 0.033 visits per flower per 30 min, *N* = 66, Table 1). The four visitor species showed different flower foraging behaviours. *B. trifasciatus* (Fig. 3C) foraged for nectar and collected pollen by grooming it into the scopae, but did not actively collect pollen from flowers. This species has a long proboscis which matches properly with the long and curved flower spur of *I. oxyanthera*. When *B. trifasciatus* entered the corolla of male-phase flowers to forage for nectar, its thoracic dorsum and wings contacted with the anthers and removed a lot of white pollen grains (Fig. 3C). Afterwards, it visited the female-phase flowers, and the thoracic dorsum and wings contacted with the stigma and deposited pollen onto the receptive stigma. *B. trifasciatus* was identified as the effective pollinator of *I. oxyanthera* (Appendix S2). Furthermore, *B. trifasciatus* groomed pollen into the corbiculae from its thoracic dorsum. Large quantities of pollen grains were found in their corbiculae and determined under a light microscope as pollen grains of *I. oxyanthera* by their shape and size. Thus, we concluded that *B. trifasciatus* is a pollen- and nectar-collecting pollinator. *B. breviceps* (Fig. 3D) has a short proboscis and sucks nectar through a hole made on the nectar spur without touching the stamen or pistil of *I. oxyanthera* (Fig. 3D).This species was identified as a nectar robber of *I. oxyanthera* (Appendix S3). It was observed that this species robbed almost all nectar from a flower in a single visit. The flower nectary is located at the tip of the nectar spur. Although the nectary can secrete nectar continuously for days (Fig. 1), it stops secreting if the nectar spur is severely damaged by nectar robbers. Butterflies (Fig. 3E) simply sucked the nectar and visited both male and female-phase flowers. Although butterflies had the possibility of pollination, they rarely visited (Table 1; Fig. 3E). We observed a few visits of honey bees to the flowers of *I. oxyanthera* (Table 1). They only collected pollen on the male-phase flowers and packed it onto their hind legs, but they did not have any contact with the receptive stigma (Fig. 3F). Honey bees were identified as pollen thieves of *I. oxyanthera* (Appendix S4). However, it is common for honey bees to collect only a part but not all pollen from a flower in a single visit.

**Table 1.** Visitors and their behaviour on flowers of *Impatiens oxyanthera*.

Visitor	Visitation rates*	Number (%) of visits^†^	Visitor type	Reward
*Bombus trifasciatus*	0.401 ± 0.042a	101 (54.301)	Pollinator	Nectar, pollen
*Bombus breviceps*	0.212 ± 0.039b	61 (32.796)	Nectar robber	Nectar
Butterflies	0.044 ± 0.014c	19 (10.215)	Pollinator	Nectar
Honeybees	0.008 ± 0.033c	5 (2.688)	Pollen thief	Pollen

*Visits per flower per 30 min (mean ± SE). Different letters show significant differences at *P* < 0.05 (GLMs).

^†^Visits of insect species. Percentage of the total visits is indicated in bracket.

### Effects of flowers removed of either pollen or nectar on pollinator behaviours

**Figure 4.**
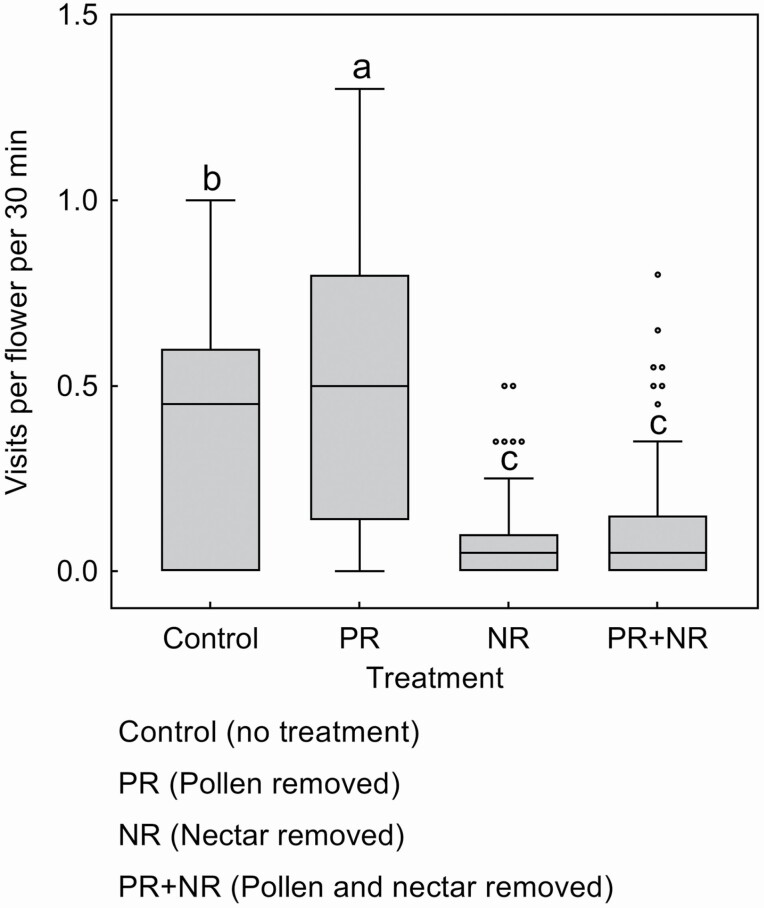
Number of pollinator *Bombus trifasciatus* visits per flower per 30 min (mean ± SE) to flowers (*N* = 380) subjected to four experimental treatments. Different lowercase letters show significant differences at *P* < 0.05 (GLMs). The box plots indicate median (mid lines), inter quartile range (boxes) and 1.5 times the inter quartile range (whiskers) as a well as outliers (points).

The GLM analysis showed that there were significant differences in the visitation rates of *B. trifasciatus* pollinator to flowers in the four experimental treatments (Wald χ^2^ = 107.581, df = 3, *P* < 0.001, Fig. 4). The visitation rate of *B. trifasciatus* to treatment 1 (control) flowers was 0.4 ± 0.04 visits per flower per 30 min (mean ± SE, *N* = 66, Fig. 4), which was significantly lower than that to treatment 2 flowers (pollen removed, 0.51 ± 0.05 visits per flower per 30 min, *P* < 0.05, *N* = 66, Fig. 4), but higher than that to treatment 3 flowers (nectar removed, 0.09 ± 0.02 visits per flower per 30 min, *P* < 0.001, *N* = 64, Fig. 4) and treatment 4 flowers (pollen and nectar removed, 0.13 ± 0.02 visits per flower per 30 min, *P* < 0.001, *N* = 64, Fig. 4). Its visitation rate to treatment 3 (nectar removed) and treatment 4 flowers (pollen and nectar removed) did not differ significantly.

**Figure 5.**
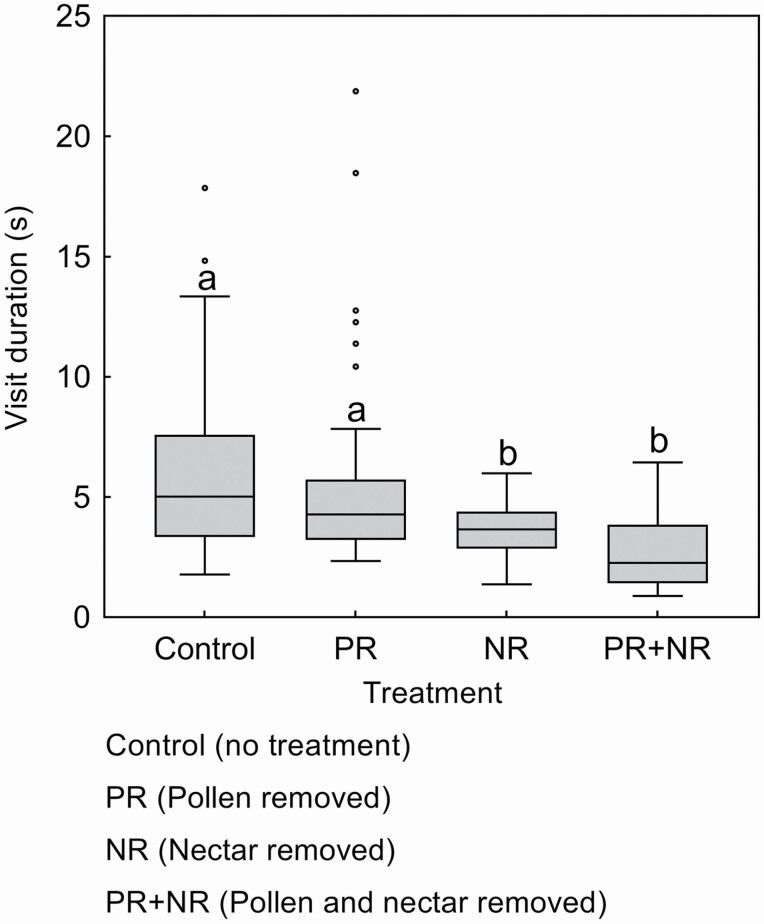
Visit duration (mean ± SE) of pollinator *Bombus trifasciatus* to flowers subjected to four experimental treatments. Different lowercase letters show significant differences at *P* < 0.05 (GLMs). The box plots indicate median (mid lines), inter quartile range (boxes) and 1.5 times the inter quartile range (whiskers) as a well as outliers (points).

Moreover, there were significant differences in the visit duration of *B. trifasciatus* to flowers among the four experimental treatments (Wald χ^2^ = 21.872, df = 3, *P* < 0.001, Fig. 5). The visit duration of *B. trifasciatus* to treatment 1 (control, 5.92 ± 0.58 s, *N* = 38, Fig. 5) and treatment 2 flowers (pollen removed, 5.78 ± 0.69 s, *N* = 39, Fig. 5) were not significantly different, but were both longer than that to treatment 3 (nectar removed, 3.63 ± 0.19 s, *P* < 0.05, *N* = 30, Fig. 5) and treatment 4 flowers (pollen and nectar removed, 2.69 ± 0.35 s, *P* < 0.001, *N* = 22, Fig. 5). There were no significant differences between its visit duration to treatment 3 (nectar removed) and treatment 4 flowers (pollen and nectar removed).

## Discussion

Our investigations on *I. oxyanthera* demonstrated that its sexual reproduction depended on pollinators and that the two floral rewards (pollen and nectar) were consumed by various visitors, including pollinators, nectar robbers and pollen thieves. Our experiments provided evidence that compared with control flowers, flowers removed of nectar attracted fewer bumblebee pollinators, supporting our hypothesis. However, our hypothesis that pollen removal would reduce pollinator visits was not supported. Instead, the flowers that contained only nectar attracted more bumblebee pollinators than the control flowers.

Flowers of *I. oxyanthera* produce a lot of pollen and nectar, and in the male phase, they provide visitors with both pollen and nectar. The pollination treatments indicated that neither spontaneous autogamy nor apomixis occurred, even though the studied species is self-compatible. Because of complete dichogamy, spontaneous autogamy was avoided. Therefore, in nature, sexual reproduction of *I. oxyanthera* depends on pollination by pollinators.

The reason why fewer visits to flowers were observed in the treatment in which the nectar was removed than in the control treatment because nectar might be the main reward for bumblebee pollinators in *I. oxyanthera*. According to our observations, the pollinator species *B. trifasciatus* only groomed a part of the pollen into the corbiculae, rather than actively collecting pollen from the male flowers of *I. oxyanthera*. Even though they probably do not want to, bumblebee pollinators have to groom the pollen because the mechanosensory input from pollen resting on the hairs of the body triggers the bumblebees to groom themselves (Thorp 2000; Lunau *et al.* 2014; Hao *et al.* 2020). Moreover, in *I. oxyanthera*, pollen may be mainly used as a visual signal for pollinators. The flowers of *I. oxyanthera* are protandrous and completely dichogamous, and we found that the nectar volume was increasingly secreted with the progress of time during the flowering phase. Flowers without anthers (i.e. without pollen) in the female phase have a greater nectar volume, and bumblebee pollinators can use the absence of pollen to identify which flowers are in the female phase and may thus have more nectar. Therefore, the removal of pollen from flowers positively affects the behaviour of bumblebee pollinators. This result is not consistent with the results of a previous study which suggested that bees visited emasculated plants less frequently than they visited non-emasculated controls of *Aloe tenuior* because native honey bees and solitary bees are the primary pollinators of this species and are mainly attracted by the presence of exposed pollen (Duffy *et al.* 2014). Pollen has a dual function: it is not only a reward for pollinators, but it also has a purpose in plant reproduction (Schlindwein *et al.* 2005; Tong and Huang 2018). Pollen placed on a pollinator’s body may be picked up by conspecific stigmas or collected by the pollinator as food (Tong and Huang 2018). In *I. oxyanthera*, we observed that bumblebee pollinators only groomed a part of the pollen grains around their bodies into the corbiculae, and the remaining pollen grains were available to conspecific stigmas when they visited the female-phase flowers.

In addition, our results showed that the pollinator species *B. trifasciatus* repeatedly visited the flowers with only the nectar reward (treatment 2), but rarely visited the flowers with only the pollen reward (treatment 3), which suggested that the foraging behaviour of *B. trifasciatus* may be negatively affected by nectar robbers but not by pollen thieves in *I. oxyanthera*. A previous study also revealed that the visitation rates of legitimate pollinators were reduced by nectar robbers in *Lonicera etrusca* (Rojas-Nossa *et al.* 2021). Besides, the reward in flowers with only pollen was not sufficient to attract bumblebee pollinators, which likely explained why the flowers produced the additional nectar reward in *I. oxyanthera*. On the contrary, plant reproductive fitness may be affected by bumblebee pollinators visiting flowers only for pollen. For example, Schlindwein (2005) found that 95.5 % of the pollen produced by the flower of *Campanula rapunculus* (Campanulaceae) was collected by pollinator bees for their offspring, 3.7 % contributed to pollination, and 0.8 % remained on the styles. Given that flowers removed of pollen were visited by more bumblebee pollinators in *I. oxyanthera*, it is possible that pollen thieves could increase the female fitness of *I. oxyanthera* by ‘stealing’ pollen reward entirely and increasing visitation of bumblebee pollinators to the flowers. We did not investigate the effects of pollen thieves on male and female fitness of plants in this study, and this should be the focus of future work.

## Supporting Information

The following additional information is available in the online version of this article—

**Appendix S1.** Raw data sets involved in this study of pollination in *Impatiens oxyanthera* including insect visits, nectar production, pollen production, fruit set and seed set under different pollination treatments, visitation rates and visit duration under four experimental treatments.

**Appendix S2.** A video involved in a pollinator *Bombus trifasciatus* is grooming pollen into corbiculae and then entering the corolla to forage for nectar.

**Appendix S3.** A video involved in a nectar robber *Bombus breviceps* is sucking nectar through a hole bitten on the nectar spur.

**Appendix S4.** A video involved in a pollen thief honey bee is collecting pollen on a male-phase flower.

plab029_suppl_Supplementary_Materials

## Data Availability

The raw data are available in **Appendix S1**.

## Notes

**Populations & Communities. Chief Editor:** Jean Burns
